# Combined lactate, base excess, and MEWS score as predictors of ICU transfer from the emergency department: a retrospective cohort study

**DOI:** 10.3389/fmed.2026.1759754

**Published:** 2026-01-20

**Authors:** Li Li, Luo Yang, Zhao Xin, Du Yao, Li xuchu, Wang Yang, Meng Qiong, Zhang Meiyuan

**Affiliations:** 1 Department of Intensive Care Unit, The International Peace Maternity and Child Health Hospital, School of Medicine, Shanghai Jiao Tong University, Shanghai, China; 2 Shanghai Key Laboratory of Embryo Original Diseases, Shanghai, China; 3 Department of Intensive Care Unit, Jiujiang University Affiliated Hospital, Jiujiang, China; 4 Department of Rehabilitation Therapy, Yunnan University of Chinese Medicine, Yunnan, China

**Keywords:** base excess, emergency department, intensive care unit, lactate, modified early warning score, mortality

## Abstract

**Objective:**

Accurate risk stratification of emergency department (ED) patients transferred to the intensive care unit (ICU) is essential for improving outcomes. The traditional Modified Early Warning Score (MEWS) alone has limited utility in predicting mortality risk. Combining MEWS with arterial blood gas parameters—lactate (Lac) and base excess (BE)—may enhance predictive performance.

**Methods:**

A retrospective cohort study was conducted involving 262 ED patients admitted to the ICU, who were divided into a non-survivor group (*n* = 83, 28-day mortality rate: 31.68%) and a survivor group (*n* = 179). Multivariable Cox regression analysis was used to identify independent predictors of 28-day mortality. Model performance was evaluated using receiver operating characteristic (ROC) curve analysis, DeLong test, net reclassification improvement (NRI)/integrated discrimination improvement (IDI) metrics, and stratified validation.

**Results:**

Multivariate Cox regression analysis showed that both Lac were independent risk factors for 28-day mortality (both *p* < 0.05). ROC curve analysis revealed that the Lac + BE + MEWS combined model achieved the highest area under the curve (AUC) of 0.819 (95% CI: 0.760–0.870), which was significantly superior to other models. The BE + MEWS model yielded an AUC of 0.805 (95% CI: 0.749–0.864), with no statistically significant difference from the Lac + BE + MEWS model (*p* = 0.098). NRI and IDI analyses indicated that both the BE + MEWS and Lac + BE + MEWS models markedly improved predictive performance (NRI: 85.9 and 83.1%, respectively; IDI: 0.249 and 0.255, respectively; both *p* < 0.001). Notably, BE conferred greater incremental value to MEWS than Lac. Stratified validation confirmed that the Lac + BE + MEWS model exhibited the best stability and risk-stratification capacity, especially in the intermediate-risk stratum (5 ≤ MEWS ≤ 8, AUC = 0.812).

**Conclusion:**

Lac and BE are independent predictors of 28-day mortality in ED patients admitted to the ICU. BE adds significantly more predictive value to MEWS than Lac. The Lac + BE + MEWS combined model demonstrates the strongest stability and optimal risk-stratification performance, particularly for patients with MEWS scores of 5–8.

## 1 Introduction

With the accelerating global aging process, the proportion of patients transferred from the emergency department (ED) to the intensive care unit (ICU) has been continuously rising (1). Literature reports indicate that as many as 46% of ICU patients were directly admitted from the ED (2). Delayed transfer of ED patients to the ICU is associated with increased mortality (3) and prolonged ICU length of stay (4), and this association becomes more pronounced as the severity of the patient’s condition increases (5). Therefore, early accurate risk stratification of critically ill patients in the ED is essential. Rational allocation of medical resources based on this stratification has thus become a key link in optimizing the management pathway of critically ill patients and improving clinical outcomes. The Modified Early Warning Score (MEWS), a commonly used condition assessment tool in the ED, has limited predictive efficacy for critically ill patients (6), which often leads to delayed transfer of high-risk patients to the ICU or improper resource allocation (7, 8). Thus, identifying rapid and efficient biomarkers to complement MEWS and improve predictive accuracy is essential for developing more effective prognostic models and this remains an urgent challenge in emergency critical care.

Lactate (Lac) is the end product of anaerobic metabolism. Elevated Lac levels directly reflect tissue hypoxia and hypoperfusion, serving as a sensitive indicator of cellular energy metabolism disorders (9). As a core indicator reflecting the body’s acid–base balance status, base excess (BE) with an increased negative value indicates metabolic acidosis, indirectly reflecting tissue hypoperfusion and the severity of the patient’s condition (10). However, conclusions remain inconsistent regarding the independent predictive value of Lac and BE in patients transferred from the ED to the ICU. Few studies have explored whether combining each of these biomarkers with MEWS can improve predictive performance, or which combination (Lac + MEWS or BE + MEWS) provides greater incremental value. Addressing this gap is essential for optimizing risk stratification strategies for emergency critically ill patients. Based on this, this study aims to achieve two core objectives: (1) To compare the predictive value of Lac and BE for 28-day mortality risk in patients transferred from the ED to the ICU; (2) To evaluate the predictive efficacy of the Lac + MEWS, BE + MEWS, and three-indicator combined models, identify the optimal model that best compensates for MEWS’ limitations, and provide evidence-based support for rapid risk stratification of emergency critically ill patients and optimal allocation of medical resources.

## 2 Materials and methods

### 2.1 Study population

Adult patients (aged ≥ 18 years) transferred from ED to ICU at the Affiliated Hospital of Jiujiang University between May 2022 and April 2024 were retrospectively enrolled. Inclusion criteria: (1) Transfer to ICU within 24 h of ED visit; (2) Lac and BE measurements, and MEWS assessment completed within 1 h of ED admission; (3) Availability of complete clinical data. Exclusion criteria: (1) Pregnant or lactating women; (2) Patients with baseline Lac/BE abnormalities caused by chronic metabolic diseases (e.g., end-stage renal disease, liver cirrhosis, chronic obstructive pulmonary disease); (3) ICU length of stay < 24 h; (4) Missing key indicators in clinical data.

### 2.2 Data collection

Relevant information was extracted from the hospital electronic medical record system: (1) Baseline data: Age, gender, comorbidities (e.g., hypertension, diabetes mellitus, coronary heart disease), and reasons for ED admission (e.g., infectious diseases, cardio-cerebrovascular diseases, digestive system bleeding); (2) Predictive indicators: Arterial blood Lac, BE (direct measurement via blood gas analyzer), and MEWS. MEWS is calculated based on 5 indicators: body temperature, heart rate, respiratory rate, systolic blood pressure, and consciousness level. Its total score ranges from 0 to 14, with higher scores meaning more severe conditions; (3) Outcome indicator: 28-day all-cause mortality. It refers to death events within 28 days of ICU transfer, including deaths in ICU and after transfer out (see Supplementary Figure 1).

### 2.3 Ethical considerations

The study protocol strictly adhered to the ethical principles of the Declaration of Helsinki and was approved by the Institutional Review Board (IRB) of Affiliated Hospital (approval number: No. JJUM20240012). Given that this was a retrospective observational study involving only secondary analysis of anonymized clinical data, with no additional patient interventions and no potential risk to patient privacy or rights, the IRB granted a waiver of informed consent. All data access and processing followed strict confidentiality protocols, and patients’ personal information was de-identified to ensure privacy and security.

### 2.4 Statistical methods

Statistical analyses were performed using Graphpad Prism 10.1.2 and R 4.5.2 software. Normally distributed continuous data were presented as mean ± standard deviation (*x* ± *s*), and compared using t-test. Non-normally distributed continuous data were expressed as median (interquartile range) [M (Q1, Q3)], and compared via Mann–Whitney *U*-test. Categorical data were presented as case number (percentage) [*n* (%)], and compared using *χ*^2^ test. Multivariate Cox regression analysis was performed to identify independent predictors of 28-day mortality. Indicators with *p* < 0.1 in univariate analysis and those with important clinical significance were included in the model. ROC curve analysis was used to evaluate the predictive value of Lac, BE, MEWS alone, as well as combined models (Lac + MEWS, BE + MEWS) for 28-day mortality. The DeLong test was used to compare differences in AUC. NRI and IDI were used to assess the predictive gain of combined models versus MEWS alone. An NRI > 0 or IDI > 0 indicated better performance of combined models. *p* < 0.05 was considered statistically significant for all analyses.

## 3 Results

### 3.1 Baseline characteristics

A total of 262 patients were enrolled. Among them, 141 (53.82%) were male and 121 (46.18%) were female, with a mean age of (69.86 ± 14.63) years. Eighty-three patients died, resulting in a 28-day mortality rate of 31.68%. Compared with survivors, non-survivors were older and had higher MEWS scores, higher Lac levels, and more negative BE values. No significant differences were found in gender, comorbidities, or causes of ED presentation between the two groups (all *p* > 0.05) (Table 1).

**Table 1:** Baseline characteristics and clinical indicators of ED patients admitted to ICU by outcome.

Variables	Total (*n* = 262)	Survival group (*n* = 179)	Death group (*n* = 83)	*p* value
Age (years)	69.86 ± 14.63	68.29 ± 15.01	73.24 ± 13.23	0.008
Male (*n* %)	141 (53.82%)	99 (55.31%)	42 (50.60%)	0.45
Comorbidities (*n* %)				
HTN	163 (62.21%)	114 (63.69%)	49 (59.04%)	0.86
DM	157 (59.92%)	119 (66.48%)	38 (45.78%)	0.13
Emergency visit reasons (*n* %)				
ID	58 (22.14%)	33 (18.44%)	25 (30.12%)	0.052
CCVD	54 (20.61%)	38 (21.23%)	16 (19.28%)	0.74
GIB	66 (25.19%)	53 (29.61%)	13 (15.66%)	0.09
MT	26 (9.92%)	18 (10.06%)	8 (9.64%)	0.92
Stroke	27 (10.31%)	16 (8.94%)	11 (13.25%)	0.24
Others	31 (11.83%)	21 (11.73%)	10 (12.05%)	0.99
MEWS	6.01 ± 2.31	5.72 ± 1.91	6.78 ± 2.88	0.003
Lac (mmol/L)	4.73 ± 5.13	3.29 ± 3.54	7.83 ± 6.51	<0.001
BE (mmol/L)	−6.20 ± 8.80	−3.04 ± 6.44	−13.01 ± 9.37	<0.001

HTN, Hypertension; DM, Diabetes Mellitus; ID, Infectious Diseases; CCVD, Cardio-Cerebrovascular Diseases; GIB, Gastrointestinal Bleeding; MT, Multiple Trauma; MEWS, Modified Early Warning Score; Lac, Lactate; BE, Base Excess.

### 3.2 Multivariate cox regression analysis

Multivariate Cox regression analysis was performed after adjusting for age, MEWS score, Lac, BE, infection, bleeding, and gender (Table 2). The model showed good fit (AIC = 724.3). Each 1-year increase in age was associated with a 2.0% higher risk of death (HR = 1.020, 95% CI 1.003–1.040). For Lac, each 1 mmol/L increase raised the death risk by 10.2% (HR = 1.102, 95% CI 1.043–1.166). For BE, each 1 mmol/L decrease increased the death risk by 5.5% (HR = 0.945, 95% CI 0.917–0.975). Infection increased the death risk by 81% (HR = 1.810, 95% CI 1.024–3.314), and bleeding by 100.5% (HR = 2.005, 95% CI 1.142–3.779). Gender and MEWS score were not independent predictors of death (both *p* > 0.05).

**Table 2:** Multivariate cox regression analysis of risk factors for mortality in ED patients admitted to ICU (*n* = 262).

Variables	*β*	*SE*	HR (95% CI)	*P*
Age	0.02	0.009	1.020 (1.003–1.040)	0.02
MEWS	−0.021	0.045	0.979 (0.895–1.068)	0.64
Lac	0.098	0.028	1.102 (1.043–1.166)	<0.01
BE	−0.057	0.016	0.945 (0.917–0.975)	<0.01
Infection	0.594	0.299	1.810 (1.024–3.314)	0.04
Bleeding	0.696	0.303	2.005 (1.142–3.779)	0.02
Sex	−0.035	0.223	0.965 (0.622–1.496)	0.87

HR, hazard ratio; CI, confidence interval; β, regression coefficient; SE, standard error; MEWS, Modified Early Warning Score; Lac, Lactate; BE, Base excess.

### 3.3 ROC curve analysis and bootstrap validation

**Figure 1:**
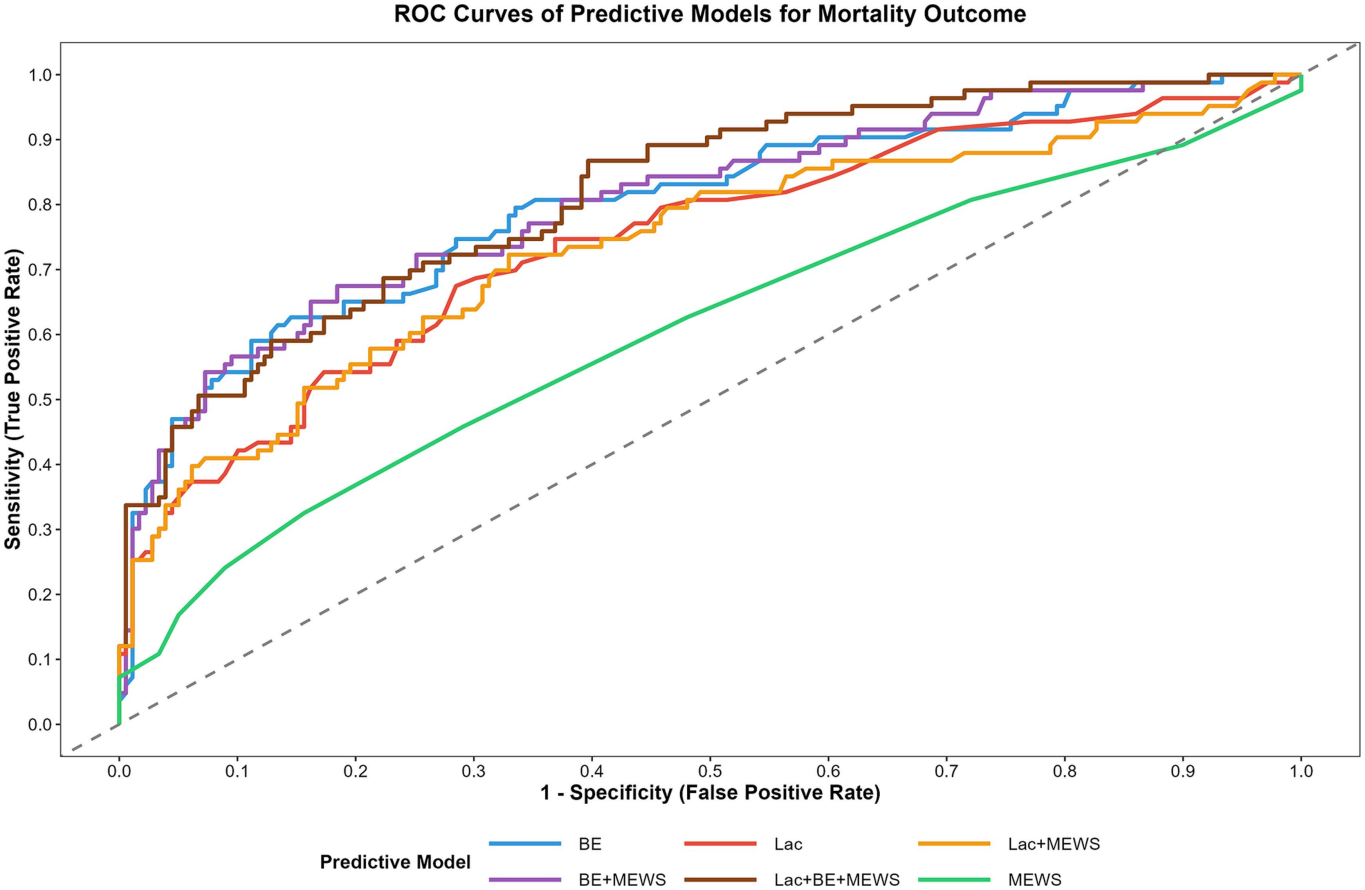
ROC curves of different predictive models for mortality outcome in ED patients admitted to ICU. The diagonal dashed line represents the reference line (AUC = 0.5, no predictive ability). The curve closer to the top-left corner indicates better predictive performance. BE, Base Excess; Lac, Lactate; MEWS, Modified Early Warning Score. The model names represent combinations of these indicators.

**Figure 2:**
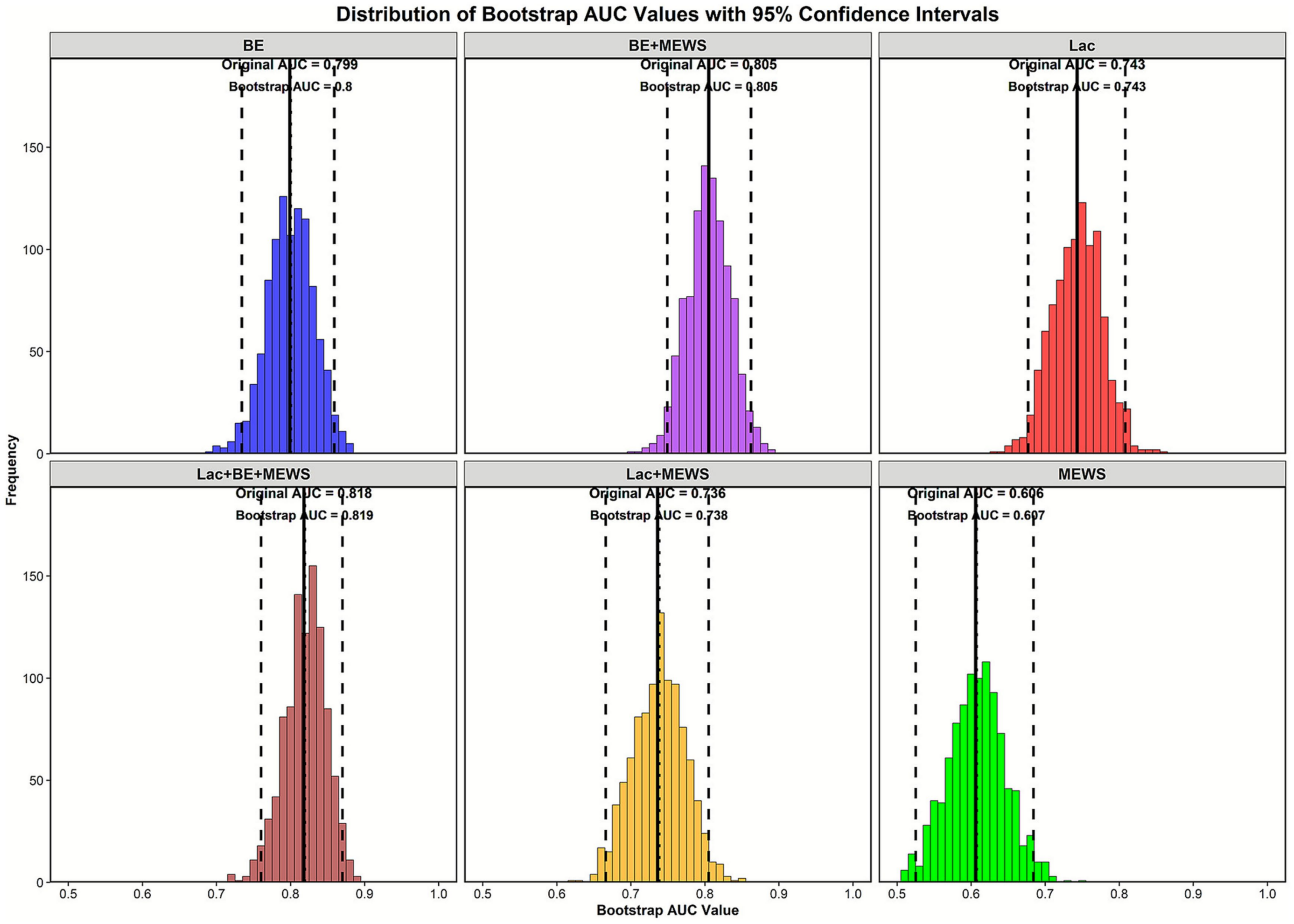
Bootstrap distributions of AUC values for multiple predictive models. Each subplot shows the bootstrap distribution of AUC values for a specific predictive model (e.g., BE, Lac + MEWS). In each plot: the dotted-line indicates the original AUC, the solid black line indicates the bootstrap mean AUC, and the dashed-line area represents the 95% confidence interval (CI) of the bootstrap AUC. A more concentrated distribution indicates greater stability of the model’s predictive performance.

The DeLong tests were used to compare the performance of single and combined models based on Lac, BE, and MEWS. The AUC of BE alone was 0.799 (95% CI 0.734–0.859), and its performance was not significantly different from that of the Lac + BE + MEWS model (*p* = 0.156) (Table 3, Figure 1). For Lac alone, the AUC was 0.743 and the Lac + MEWS combined model (AUC = 0.736) were significantly inferior to the Lac + BE + MEWS model (*p* = 0.018 and 0.008, respectively). MEWS alone exhibited the lowest AUC (0.606) and suggesting it may not be sufficient for standalone clinical use. The BE + MEWS combined model achieved an AUC of 0.805 (95% CI 0.749–0.864), which showed no significant difference from the Lac + BE + MEWS model (*p* = 0.098). It can thus serve as an efficient alternative. The Lac + BE + MEWS model had the highest AUC (0.819, 95% CI 0.763–0.872) and was significantly better than Lac alone, Lac + MEWS, and MEWS alone (all *p* < 0.001). Bootstrap validation (n = 1,000) confirmed that the AUC estimates of all models were stable (Figure 2).

**Table 3:** Comparison of the predictive performance of various indicators for mortality risk (DeLong method, *n* = 262).

Model	AUC 95%CI	vs. Lac (*p*-value)	vs. BE (*p*-value)	vs. Lac + MEWS (*p*-value)	vs. BE + MEWS (*p*-value)	vs. Lac + BE + MEWS (*p*-value)	Bootstrap AUC 95%CI
Lac	0.743 (0.676–0.81)	—	—	—	—	—	0.743 (0.677–0.808)
BE	0.799 (0.738–0.859)	0.159	—	—	—	—	0.8 (0.734–0.859)
Lac + MEWS	0.736 (0.667–0.805)	0.007	0.118	—	—	—	0.738 (0.666–0.805)
BE + MEWS	0.805 (0.746–0.864)	0.062	0.583	0.059	—	—	0.805 (0.749–0.862)
Lac + BE + MEWS	0.818 (0.763–0.872)	0.018	0.156	0.008	0.098	—	0.819 (0.76–0.87)
MEWS	0.606 (0.529–0.682)	<0.001	<0.001	<0.001	<0.001	<0.001	0.607 (0.525–0.684)

AUC, area under the receiver operating characteristic curve; CI, confidence interval. *p*-values are derived from pairwise comparisons of ROC curves (DeLong’s test).

### 3.4 Net reclassification and integrated discrimination improvement

To quantify the incremental value of combining Lac or BE with MEWS, we calculated the NRI and IDI relative to MEWS alone. All combined models significantly improved predictive performance compared with MEWS alone (all *p* < 0.001). Adding Lac to MEWS yielded a NRI of 56.1% (95% CI 35.3–77.0%) and an IDI of 0.152 (95% CI 0.096–0.209). The BE + MEWS model achieved the highest NRI of 85.9% (95% CI 64.5–106.4%) and an IDI of 0.249 (95% CI 0.188–0.312), whereas the Lac + BE + MEWS model produced an NRI of 83.1% (95% CI 61.9–105.9%) and an IDI of 0.255 (95% CI 0.189–0.320). Thus, both BE-based combinations conferred the greatest gain in reclassification and discriminative capacity (Table 4).

**Table 4:** Comparison of NRI and IDI for predictive models using MEWS as reference.

Model	NRI 95% CI	NRI *p*-value	IDI 95% CI	IDI *p*-value
Lac + MEWS	0.561 (0.353–0.770)	<0.001	0.152 (0.096–0.209)	<0.001
BE + MEWS	0.859 (0.645–1.064)	<0.001	0.249 (0.188–0.312)	<0.001
Lac + BE + MEWS	0.831 (0.619–1.059)	<0.001	0.255 (0.189–0.320)	<0.001

NRI, net reclassification improvement; IDI, integrated discrimination improvement. Values in parentheses are 95% confidence intervals. *p*-values were calculated by the bootstrap method with a two-sided α of 0.05.

### 3.5 Predictive performance across MEWS risk strata

When patients were subdivided into low- (MEWS ≤ 4), intermediate- (MEWS 5–8), and high-risk (MEWS ≥ 9) categories, the Lac + BE + MEWS model consistently achieved an AUC ≥ 0.787 in each stratum, indicating stable performance (Table 5). In the intermediate-risk cohort (*n* = 158), its AUC reached 0.812 (95% CI 0.737–0.886), outperforming MEWS alone (*p* < 0.001) and Lac + MEWS (*p* = 0.042), while remaining comparable with BE + MEWS (AUC 0.798; *p* = 0.279). In the high-risk group (*n* = 62), all combined models produced AUC ≥ 0.845, with the Lac + BE + MEWS model attaining the highest value of 0.859 (95% CI 0.740–0.978). In the low-risk stratum (*n* = 42), both the Lac + BE + MEWS model and BE + MEWS registered an AUC of 0.787, but neither improvement reached statistical significance compared with MEWS or Lac + MEWS (all *p* > 0.07). Consequently, the Lac + BE + MEWS algorithm offers the most robust and uniform prognostic power across the entire spectrum of ED-to-ICU patients, particularly within the clinically pivotal intermediate-risk population.

**Table 5:** Comparison of AUCs across predictive models within MEWS strata.

MEWS Strata	Model	AUC (95% CI)	Vs. MEWS (*p*-value)	Vs. Lac + MEWS (*p*-value)	Vs. BE + MEWS (*p*-value)
5 ≤ MEWS≤8	MEWS	0.544 (0.449–0.639)	—	—	—
	Lac + MEWS	0.722 (0.633–0.810)	0.002	—	—
	BE + MEWS	0.798 (0.715–0.881)	<0.001	0.15	—
	Lac + BE + MEWS	0.812 (0.737–0.886)	<0.001	0.042	0.279
MEWS≥9	MEWS	0.620 (0.442–0.799)	—	—	—
	Lac + MEWS	0.847 (0.721–0.973)	0.025	—	—
	BE + MEWS	0.845 (0.720–0.970)	0.051	0.978	—
	Lac + BE + MEWS	0.859 (0.740–0.978)	0.033	0.795	0.383
MEWS≤4	MEWS	0.624 (0.473–0.774)	—	—	—
	Lac + MEWS	0.627 (0.451–0.804)	0.977	—	—
	BE + MEWS	0.787 (0.660–0.914)	0.161	0.095	—
	Lac + BE + MEWS	0.787 (0.661–0.914)	0.157	0.07	0.966

AUC, area under the receiver operating characteristic curve; CI, confidence interval. *p*-values were calculated using DeLong’s test to assess differences in AUC between the current model and the reference model indicated in the column header.

## 4 Discussion

Against the backdrop of insufficient predictive efficacy of MEWS in ED-to-ICU transferred patients, this study was the first to explore the predictive value of Lac and BE alone and in combination with MEWS for 28-day mortality risk in these patients. The core conclusions are as follows: (1) Both Lac and BE are independent risk factors for 28-day mortality in these patients; (2) BE provides significantly greater predictive gain to MEWS than Lac; (3) The Lac + BE + MEWS model has the best stability and risk stratification ability, particularly in the intermediate-risk population with MEWS scores of 5–8.

Arterial blood gas (ABG) analysis is a core method for evaluating acid–base balance and tissue metabolic status in critically ill patients. As a direct biomarker of tissue hypoxia, elevated Lac levels have been confirmed by numerous studies to be associated with poor prognosis in critically ill patients (11, 12). However, critically ill patients with normal Lac levels are common in clinical practice. Sauer CM et al. enrolled 4,861 patients and found that 47% of them had normal Lac levels, although their 28-day mortality rate was lower than that of the high-Lac group, it was still as high as 17% (13). Kim et al. (14) also observed 617 cases with normal Lac levels among 2032 sepsis patients. These findings suggest that relying solely on Lac for risk assessment may miss some high-risk individuals, which is not conducive to accurate risk stratification.

While Lac has demonstrated prognostic value in critically ill patients, its limitations in identifying high-risk individuals—especially in heterogeneous ED-to-ICU populations—cannot be overlooked. In contrast, BE, as a comprehensive indicator reflecting the body’s overall acid–base balance, showed superior predictive performance for 28-day mortality in our study. BE is a core indicator reflecting the overall acid–base balance status of the body and is often used as an independent predictor of mortality in critically ill patients (15, 16). Inconsistent with some previous studies that concluded Lac has superior predictive value (17), this study found that BE outperformed Lac in predictive efficacy. The AUC of BE alone (0.799) was significantly higher than that of Lac (0.743), indicating that BE is more accurate in identifying patients at risk of death. A possible reason is that the patients enrolled in this study covered a variety of etiologies (infection, gastrointestinal bleeding, cardio-cerebrovascular diseases, etc.), rather than focusing solely on specific populations such as sepsis. As a comprehensive indicator of acid–base balance, BE can better reflect tissue hypoperfusion and metabolic disorders caused by different etiologies.

Traditional EWS, such as MEWS, are commonly used rapid assessment tools in the ED. They rely on macroscopic vital signs and consciousness level, and have limitations in predicting mortality risk (18). In this study, the AUC of MEWS alone was only 0.606, confirming its insufficient predictive efficacy. Therefore, supplementing traditional scores with laboratory indicators has achieved good results. For example, Unal Cetin et al. (19) used the lactate-albumin ratio to improve the comprehensive predictive value of MEWS for 30-day mortality in ICU sepsis patients. Andersson et al. (20) found that in emergency hospitalized patients with suspected sepsis, elevated Lac increased the identification rate of non-survivors from 48 to 68%, and from 77 to 85% in the NEWS ≥7 group. Consistent with these findings, our study further confirmed that combining acid–base indicators (Lac/BE) with MEWS can significantly improve predictive accuracy, and we also compared the efficacy of different combinations (Lac + MEWS vs. BE + MEWS vs. Lac + BE + MEWS) to identify the optimal model, confirming that combining MEWS with the body’s acid–base indicators enhances its predictive accuracy.

Lac and BE from ABG were combined with MEWS in the present study. The results showed no significant difference in AUC between the BE + MEWS model (0.805) and the Lac + BE + MEWS model (0.819) (*p* = 0.098), and the former can serve as an efficient alternative. The Lac + BE + MEWS model maintained an AUC ≥ 0.787 across all MEWS strata (low-, intermediate-, and high-risk), and its stability was significantly superior to other models. In clinical practice, the intermediate-risk population (MEWS scores of 5–8) is the core controversial group for resource allocation. The AUC of the Lac + BE + MEWS model in this subgroup reached 0.812, which was significantly higher than that of MEWS alone (*p* < 0.001). It can more accurately distinguish high-risk individuals among the intermediate-risk population, avoiding overtreatment or insufficient intervention. Thus, it is more suitable as a preferred comprehensive assessment tool in clinical practice.

Several limitations of this study should be acknowledged first, it is a single-center retrospective study with a relatively limited sample size (262 cases). The enrolled population was from a single hospital, which may lead to selection bias, and the generalizability of the conclusions needs to be further validated by multi-center prospective studies. Second, this study only evaluated the predictive value of a single measurement of Lac and BE at admission. Dynamic monitoring of indicator changes (such as lactate clearance rate and BE recovery rate) may better reflect the progression of the patient’s condition and treatment response. Future multi-center prospective studies are needed to further verify the conclusions.

## 5 Conclusion

Both Lac and BE are independent risk factors for 28-day mortality in ED-to-ICU transferred patients. Importantly, BE provides significantly greater predictive gain to MEWS than Lac. The Lac + BE + MEWS model has the best stability and strongest risk stratification ability, and is especially suitable for the core intermediate-risk population with MEWS scores of 5–8. It provides a preferred basis for accurate clinical risk assessment and targeted intensive intervention.

## Data Availability

The original contributions presented in the study are included in the article/supplementary material, further inquiries can be directed to the corresponding author.
