# Supplementary figures and images for: Combined lactate, base excess, and MEWS score as predictors of ICU transfer from the emergency department: a retrospective cohort study
Source: Front Med (Lausanne). 2026 Jan 20;13:1759754. doi: 10.3389/fmed.2026.1759754 (PMC12864495; doi:10.3389/fmed.2026.1759754)

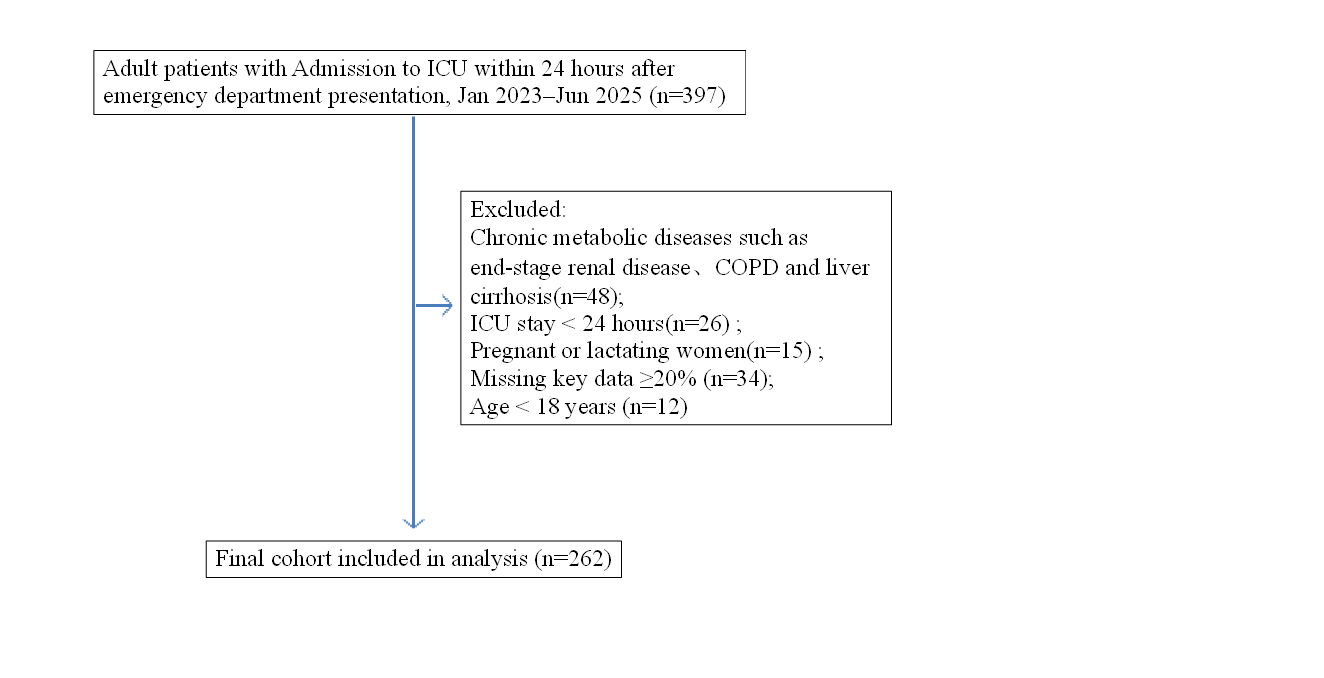

Supplement: Supplementary file 1 [file Image_1.tif]
